# Shifts in Female Facial Attractiveness during Pregnancy

**DOI:** 10.3390/ijerph17145176

**Published:** 2020-07-17

**Authors:** Dariusz P. Danel, Kasper Kalinowski, Natalia Nowak-Szczepanska, Anna Ziomkiewicz-Wichary, Anna Apanasewicz, Krzysztof Borysławski, Sławomir Kozieł, Danuta Kornafel, Pawel Fedurek

**Affiliations:** 1 Department of Anthropology, Ludwik Hirszfeld Institute of Immunology and Experimental Therapy, Polish Academy of Sciences, 53-114 Wrocław, Poland; natalia.nowak-szczepanska@hirszfeld.pl (N.N.-S.); anna.ziomkiewicz-wichary@hirszfeld.pl (A.Z.-W.); anna.apanasewicz@hirszfeld.pl (A.A.); slawomir.koziel@hirszfeld.pl (S.K.); 2 Independent researcher, 10-346 Olsztyn, Poland; kalinowski.kasper.piotr@gmail.com; 3 Department of Anthropology, Institute of Zoology and Biomedical Research, Jagiellonian University, 30-387 Krakow, Poland; 4 Department of Anthropology, Wroclaw University of Environmental and Life Sciences, 51-631 Wroclaw, Poland; krzysztof.boryslawski@upwr.edu.pl; 5 Department of Human Biology, University of Wroclaw, 50-138 Wroclaw, Poland; dankorna@wp.pl; 6 Division of Psychology, Faculty of Natural Sciences, University of Stirling, FK9 4LA Stirling, UK; pawel.fedurek@stir.ac.uk

**Keywords:** pregnancy, women, facial attractiveness, cues of fertility

## Abstract

It has been proposed that women’s physical attractiveness is a cue to temporal changes in fertility. If this is the case, we should observe shifts in attractiveness during pregnancy—a unique physiological state of temporal infertility. The aim of this study was to examine how women’s facial attractiveness changes during the subsequent trimesters of pregnancy and how it compares to that of nonpregnant women. Sixty-six pictures of pregnant women (22 pictures per trimester) and 22 of nonpregnant women (a control group) were used to generate four composite portraits, which were subsequently assessed for facial attractiveness by 117 heterosexual men. The results show considerable differences between facial attractiveness ratings depending on the status and progress of pregnancy. Nonpregnant women were perceived as the most attractive, and the attractiveness scores of pregnant women decreased throughout the course of pregnancy. Our findings show that facial attractiveness can be influenced by pregnancy and that gestation, even at its early stages, affects facial attractiveness. Considerable changes in women’s physiology that occur during pregnancy may be responsible for the observed effects.

## 1. Introduction

Women’s fertility, broadly defined as the probability of successful fertilization after sexual intercourse, changes considerably during the menstrual cycle. The lowest probability of conception occurs during menstruation, while the highest probability occurs during ovulation [1,2]. Consequently, men’s ability to detect the fertile phase of the menstrual cycle can be essential for reproductive success.

However, unlike in many animal species, where estrus is conspicuously manifested, ovulation in women is considered to be concealed and only detectable through the use of subtle cues [3]. Human markers of ovulatory status include subtle changes in voice, scent, and behavior, as well as in physical appearance (for a review, see [4]). Cyclic fluctuations in body asymmetry (i.e., women’s bilateral soft tissues become more symmetrical around ovulation [5,6]) and waist-to-hip ratio (i.e., lower values around midcycle [7]) are examples of morphological signs of the current reproductive status.

Temporary changes in fertility can be displayed by women’s faces. For example, women’s faces are perceived as more attractive in the follicular phase (i.e., the phase with a high conception probability) of the menstrual cycle [8,9,10,11]. In addition, when the probability of conception is high, women’s faces are perceived as more healthy, sexy, feminine, sociable, trustworthy, caring, likeable, and accessible as a potential date partner than in the nonfertile luteal phase of the menstrual cycle [9,10,11,12]. Nonetheless, these results have been recently challenged by studies demonstrating no association between menstrual cycle patterns and facial attractiveness (e.g., [13,14]; also, see [15], that analyzed particular facial attractiveness markers). Pregnancy is another fertility-related factor that can affect women’s facial beauty. During this period of approximately 40 weeks of “infertility”, women should be less physically attractive and less preferred by men due to the null probability of current reproduction. This hypothesis was recently examined by Prokop, Zvaríková, Zvarík, and Fedor [16]. In their experimental study, nonpregnant women were asked to wear a silicone abdomen underneath a t-shirt to simulate the third trimester of the pregnancy. Then, men’s ratings of the full-body attractiveness of nonpregnant women, and the same women with the artificially enlarged abdomen (putatively pregnant), were compared. The study found that putatively pregnant women were less preferred by men and concluded that pregnancy decreased women’s physical attractiveness.

Prokop et al.’s [16] results support the hypothesis that pregnancy has a negative influence on women’s physical attractiveness. Their experiment, however, focused only on one evident sign of pregnancy, i.e., abdomen size, and simulated only the third trimester of pregnancy. This raises the question of whether a similar effect of pregnancy on physical attractiveness can be observed earlier in pregnancy, particularly when salient pregnancy-dependent changes in body size and shape are not yet present. 

In this study, we focused on women’s faces and examined men’s perceptions of four composite portraits constructed from individual pictures of women who were either nonpregnant or in one of the three consecutive trimesters of pregnancy. Building on the findings of Prokop et al. [16], we hypothesized that facial attractiveness of women during pregnancy would be lower than that of nonpregnant women. Furthermore, we examined whether the decrease in facial attractiveness is also observed during the early stages of pregnancy, where other cues of pregnancy such as an expanded abdomen are either absent or undetectable.

## 2. Material and Methods

### 2.1. Ethics Statement

The study design was approved by the Bioethics Committee at Wroclaw Medical University (approval number: KB/854/2010). All participants gave informed written consent to participate in our study.

### 2.2. Stimuli—Composite Portraits

Facial photographs of 136 healthy pregnant adult women in different stages of pregnancy were taken from patients at a gynecological clinic in the Regional Specialist Hospital in Wrocław, Poland. All women were Caucasian and lived in Poland. The pictures were taken whilst the women were waiting for routine pregnancy check-ups. The pictures were taken in a separate room, under natural daylight conditions, using a Sony Alfa 390 digital camera with an external flash lamp. The women were asked to pin back their hair (if necessary), to keep their facial expressions neutral (no smile), and to sit straight on a chair facing the tripod which was placed about 1.5 m away from the subject. Prior to taking the picture, the camera operator adjusted the position of the subject’s head to the Frankfurt plane.

A questionnaire was used to ask the women about the status (pregnant/nonpregnant) and progress (week) of pregnancy. The onset of pregnancy was considered as the first day of the last menstrual period and was established by the gynecologist. Only women who reported no problems with their pregnancy (e.g., no pathologies) were included in the analysis. From this pool, we randomly drew trimester groups (n = 22) with photographs of each of the women: women who were pregnant for up to 13 weeks (I trimester), women between the 14th and 26th week of pregnancy (II trimester), and women who were pregnant for 27 weeks or more (III trimester). The number of women in each group equaled the number of nonpregnant women (control group; n = 22) that participated in the study. Nonpregnant women were recruited and photographed either in a hospital (individuals having standard gynecologist visits) or outside the hospital in similar indoor light conditions by the same photographer and using the same photographic methodology, equipment, and set-up (note that we did not use the color reference cards (ColorChecker patches); therefore, we were not able to formally compare the lighting and photographic conditions or to measure color values in the images). There were no statistically significant differences between the ages of the women in each group (details: Table 1).

**Figure 1:**
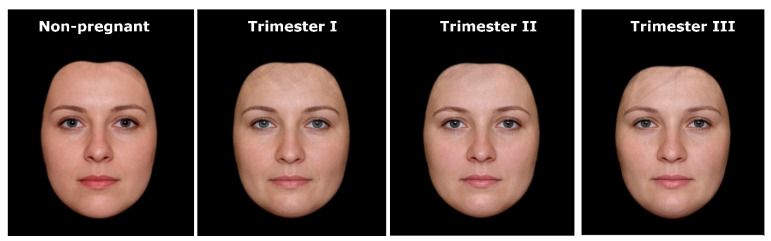
Stimuli used in the study—composite portraits of women’s faces in relation to pregnancy status and progress.

In order to construct composite portraits of pregnant and nonpregnant women, we used the face-processing software Psychomorph [17,18], which is commonly used in face averaging (e.g., [9,19,20,21,22,23,24,25]). First, a template of 179 points (landmarks) was applied to each photo to reflect the morphological diversity of women’s faces. Then, the faces in each group were digitally averaged. The resulting composite portraits were prepared by calculating the average spatial location of each landmark and by averaging the color information (red/green/blue) for each pixel (further details on the averaging procedures can be found in [17]). This procedure reveals characteristics that are consistent for a given set of pictures and conceals any individual variations of the processed faces. To avoid blurring and to create realistic images with clear facial details (e.g., wrinkles, natural skin structure), the averaging was completed with texture processing using wavelet analysis [18,26]. Finally, to expose differences in facial morphology, we used black masks to cover the ears, hair, and neck of the averaged portraits (cf. [20,24,27]). As a result, we obtained four composite pictures showing the average face shape of nonpregnant and pregnant women in trimesters I, II, and III (Figure 1).

### 2.3. Ratings of Composite Pictures

Four composite portraits were assessed for facial attractiveness by 117 heterosexual adult Caucasian men living in Poland (age range 20–70; mean = 41.15; SD = 13.86). The ethnic and populational homogeneity of both stimuli and the raters limited the potential influence of cross-cultural settings on face preferences (see [20] and citations therein). The assessed portraits had a 15 × 10 cm format and were presented separately in a random order to each individual. The attractiveness of individual faces was assessed on a 1–7 scale, where 1 represented a ”completely unattractive” face and 7 represented a “very attractive” face (cf. [12,14,23]). The ratings given for each of the portraits were averaged; thus, the final score for each individual picture denotes the mean facial attractiveness scored by the judges.

### 2.4. Statistical Analysis

We used repeated measures (within-subject) Analysis of Variance (ANOVA), and Tukey’s Honest Significant Difference (HSD) post hoc test to analyze differences in the mean attractiveness ratings of nonpregnant and pregnant women’s portraits (including women in all three trimesters of pregnancy). Mauchly’s test of sphericity indicated that the assumption of sphericity was not violated (χ^2^(5) = 8.92, *p* = 0.11). Since the basic inspection of the results indicated a regular decrease in the mean attractiveness scores, we additionally employed a planned contrast analysis to test for possible linear trends in our results. The alpha level for significance was set at 0.05. Analyses were carried out in STATISTICA, version 12 (data analysis software, www.statsoft.com). See Data S1 in Supplementary Material for the dataset used in the analysis.

## 3. Results

**Figure 2:**
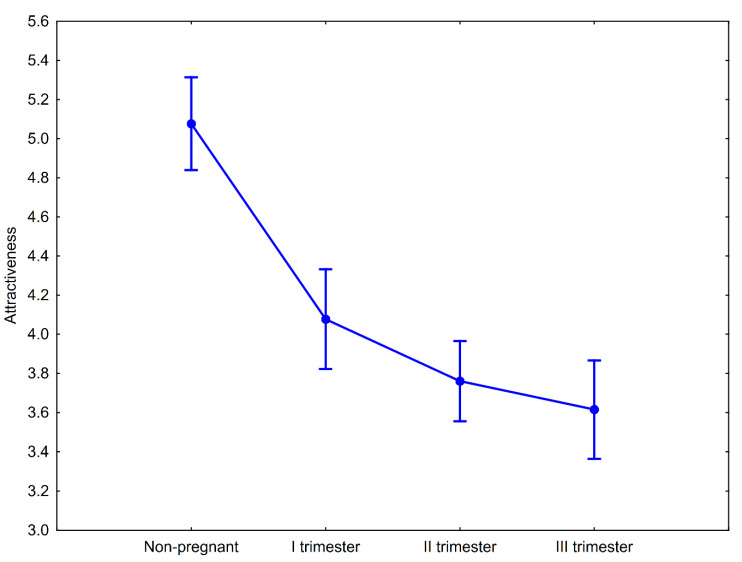
Mean attractiveness ratings of nonpregnant and pregnant women at the three stages of pregnancy. Whiskers represent 95% confidence intervals.

There were significant differences between attractiveness ratings of women’s portraits depending on their pregnancy status (F(3, 348) = 39.33, *p* < 0.00001, η_p_^2^ = 0.25). Men assessed the nonpregnant women as the most attractive and the attractiveness scores of the pregnant women decreased as the stage of pregnancy increased (Figure 2). Post hoc pairwise comparisons with Tukey’s HSD test showed that differences in facial attractiveness observed between nonpregnant and pregnant women (in the I, II, and III trimesters, separately) were significant (Table 2). The differences in attractiveness of pregnant women were significantly different only between women in trimester I and trimester III (Table 2). A formal contrast test for linear trends showed that the downward tendency in attractiveness scores in relation to pregnancy progress is significant (F(1, 116) = 88.25, *p* < 0.00001; Figure 1). Compared to the nonpregnant condition, the I trimester of pregnancy was associated with a 19.69% decline in perceived facial attractiveness. In the II and III trimesters of pregnancy, the magnitude of the decline compared to the nonpregnant condition was 25.98 and 28.74%, respectively.

## 4. Discussion

In this study, we investigated whether pregnancy is related to changes in female facial attractiveness. We found that men perceived composite portraits of pregnant women as less attractive than that of nonpregnant women. Furthermore, when compared to nonpregnant women, there was a significant decrease in the attractiveness of pregnant women, even at early stages of pregnancy, and that trend continued until the final trimester.

We showed that pregnancy was negatively related to women’s facial attractiveness, supporting Prokop et al.’s [16] findings that pregnancy decreases women’s physical attractiveness. However, in contrast to Prokop et al.’s [16] study, where pregnancy was artificially simulated by wearing a conspicuous silicone abdomen, we found that genuine pregnancy can lead to significant changes in female physical attractiveness. Furthermore, while the previous study focused only on the putative effects of late pregnancy (i.e., the third trimester) on physical attractiveness [16], our more comprehensive approach showed that the decrease in attractiveness takes place gradually throughout the whole pregnancy. Lastly, by focusing on faces, we demonstrated that the decline in attractiveness during pregnancy is not limited to body shape but might be also exhibited by more subtle changes in facial appearance.

In a broader perspective, a decrease in perceived attractiveness during pregnancy resonates with the hypothesis that women’s physical beauty mirrors current fertility status (cf. [16]). A similar attractiveness–fertility link has been reported by previous studies on temporal changes in facial attractiveness during the menstrual cycle. Faces were perceived as more physically attractive when women were in the fertile phase of the menstrual cycle [8,10,12]. Considering that this effect has been recently challenged [13], our results provide a new insight into the ongoing debate on this particular research topic by implying that women’s facial appearance, since it is modulated by pregnancy, may serve as a cue for fertility.

Since changes in women’s facial attractiveness reflect changes in both female fertility across the menstrual cycle and pregnancy status, these effects are likely mediated by steroid sex hormones. However, previous research on nonpregnant women examining the effects of estrogen and progesterone on women’s facial attractiveness produced inconclusive results. Whereas Law Smith et al. [12] found a positive relationship between the levels of estrogen and ratings of facial attractiveness, Puts et al. [28] demonstrated that it is the progesterone concentrations that are negatively associated with ratings of women’s facial beauty. More recently, Jones et al. [14] did not find convincing evidence for the link between women’s facial attractiveness and concentrations of estradiol or progesterone. Nonetheless, both Puts et al. [28] and Jones et al. [14] suggested that the interaction between these two sex steroids might, to some extent, affect women’s attractiveness.

The lack of unambiguous support for the hypothesis that cyclic fluctuations of women’s facial attractiveness have hormonal underpinnings might also bring into question the hypothesis that observed changes reflect current fertility status. It has been argued, for example, that the hormone–attractiveness relationships observed across individual menstrual cycles are merely functionless byproducts of within-women periodic hormonal fluctuations [14] or simply reflect interindividual correlations between levels of sex steroids and attractiveness rather than the current fertility status per se [29,30].

Regardless of the functional meaning and the mechanism underlying shifts in facial beauty as a function of menstrual cycle, woman’s facial attractiveness might be sensitive to physiological changes related to pregnancy. This is because both fertilization and gestation are associated with drastic changes in women’s physiology, including a steady rise in progesterone and estrogen concentrations observed throughout the pregnancy [31,32,33], which in turn might affect facial appearance.

It is also possible that the drop in women’s facial attractiveness observed in this study arises due to processes other than changes in sex steroid levels during pregnancy. Pregnancy, for example, is associated with a significant increase in body weight and accumulation of maternal fat tissue (see [34,35] and citations therein). Importantly, the rise in body weight and fat content is observed already during the first trimester of pregnancy [36,37,38]. Recent studies linked perceived female facial attractiveness to body and facial adiposity [27,39,40,41] and demonstrated that excessive fatness decreases ratings of women’s attractiveness. It is thus possible that the changes in fatness during pregnancy that potentially occurred in our study subjects affected the ratings of their facial attractiveness—an aspect that should be explored by future studies.

Another possible mechanism for the observed decrease in facial attractiveness could be associated with the modulation of the immune system observed during pregnancy [42]. It has been proposed that immune defense mechanisms are altered during pregnancy to facilitate embryo implantation, successful fetal development, and parturition. The modulation of the maternal immune system is under the control of the fetus and placenta and leads to prolonged proinflammatory and anti-inflammatory periods [42]. Oberzaucher and Grammer [43] suggested that facial attractiveness is a cue of immune resistance. According to this view, being attractive is a sign of the effectiveness of the immune system in any given environmental conditions. Consequently, a decrease of perceived facial attractiveness observed during pregnancy may indicate the fragility of the immune system during this period. However, evidence for the link between female attractiveness and immune functioning is equivocal. While several studies show that more attractive women are healthier [44,45], others did not find this effect [46]. Furthermore, the immune function markers appear to be unrelated to women’s facial beauty [27,40,46]. It is also important to note that, according to our knowledge, the link between attractiveness and the condition of the immune system in pregnant women has not been directly examined. Further studies are needed to establish whether women’s attractiveness reflects changes in immunity experienced during pregnancy.

Finally, the patterns reported in our study might be related to cortisol. The basal levels of salivary cortisol begin to rise steadily during the first trimester of gestation; by late pregnancy, they reach levels that are more than 2–3-fold the prepregnancy levels [47,48,49]. This rise in cortisol facilitates glucose inflow, which is necessary to support both fetal development and maternal immunosuppression. Cortisol levels as physiological markers of stress and immunosuppression in the context of attractiveness have been examined by previous studies, however with mixed results. While some studies [50,51] found no relationship between cortisol levels and women’s facial attractiveness, a negative association has also been reported between these two variables [40]. Although this remains to be investigated, the drop in perceived facial attractiveness in the course of pregnancy may thus be related to the increase in cortisol levels that occurs during this period.

Our study, which we consider as a promising starting point in this line of research, has several limitations. One limitation is related to the cross-sectional nature of our stimuli—the four composite portraits used in the experiment were based on four different groups of women. Future studies should replicate our results using a longitudinal within-subject design, where the subjects are monitored prior to and during pregnancy. Such an approach would also allow for precise measurements of potential pregnancy-related changes in particular markers of facial attractiveness such as averageness, symmetry, sexual dimorphism, and adiposity (reviewed in [52,53]). However, due to the design of our study, these processes could not be directly investigated since potential differences in specific facial measurements may result from between-individual differences rather than from longitudinal changes in women’s faces. Similar shortcomings (faces of different individuals in each group of women) probably also bias perceptual ratings of the facial attractiveness markers regarding individual faces. Moreover, visual evaluations of facial attractiveness markers such as symmetry, averageness, femininity, or adiposity, as well as other perceived facial qualities, are usually significantly correlated with attractiveness and with each other (e.g., [54,55,56,57,58]). Consequently, conclusions from ratings of potential pregnancy-related changes in attractiveness and its markers would likely be limited. Nonetheless, we encourage future studies to use a longitudinal approach to investigate the above processes in detail. In addition, the putative role of hormones and the immune system in the observed phenomena, that was not addressed by our study, might significantly affect women’s skin pigmentation and vasculature and cause other dermatological changes [59,60,61]. Indeed, skin condition is widely recognized as an important determinant of facial attractiveness (reviewed in [52]). Therefore, its significance for the attractiveness of pregnant women should be examined by future studies, ideally in standardized laboratory settings that would allow precise monitoring of changes in skin texture and coloration over time.

## 5. Conclusions

Our results show that women’s facial attractiveness reflects pregnancy-related fertility status and as such may act as a cue of pregnancy, even during the onset of gestation when other salient cues, such as expanded abdomen, are absent. This phenomenon may be associated with the substantial physiological changes occurring during pregnancy—an aspect that should be explored by future studies.

## Figures and Tables

**Table 1:** Age of women in each study group.

Study Group	N	Mean	SD
**Nonpregnant**	22	28.18	3.78
**I Trimester**	22	30.73	5.37
**II Trimester**	22	29.77	5.06
**III Trimester**	22	28.81	3.81
**Age Difference:**	Kruskal–Wallis test: H(3, N = 88) = 2.85, *p* = 0.42		

**Table 2:** Post hoc Tukey’s HSD pairwise comparisons of attractiveness ratings.

		Pairwise Comparisons: *p*-Values		
Stimuli		T1	T2	T3
	Attractiveness: Mean (SD)			
**Nonpregnant (NP)**	5.08 (1.294)	<0.00001	<0.00001	<0.00001
**I Trimester (T1)**	4.08 (1.391)		0.14	0.01
**II Trimester (T2)**	3.76 (1.119)			0.76
**III Trimester (T3)**	3.62 (1.370)			

## Supplementary Materials

The following are available online at https://www.mdpi.com/1660-4601/17/14/5176/s1, Data S1: Dataset used in the study.
